# Detecting *Schistosoma mansoni* infections among pre-school-aged children in southern Ghana: a diagnostic comparison of urine-CCA, real-time PCR and Kato-Katz assays

**DOI:** 10.1186/s12879-020-05034-2

**Published:** 2020-04-22

**Authors:** Samuel Armoo, Lucas J. Cunningham, Suzy J. Campbell, Frank T. Aboagye, Freda K. Boampong, Buhari A. Hamidu, Mike Y. Osei-Atweneboana, J. Russell Stothard, Emily R. Adams

**Affiliations:** 1 Biomedical and Public Health Research Unit, Council for Scientific and Industrial Research - Water Research Institute, Council Close, Accra, Ghana; 2 Department of Parasitology, Liverpool School of Tropical Medicine, Liverpool, L3 5QA UK; 3 Evidence Action, Deworm the World Initiative, Washington, DC USA

**Keywords:** Schistosomiasis, Circulating cathodic antigen, Kato-Katz, *Schistosoma mansoni*, Diagnostic, Real-time PCR Taqman®

## Abstract

**Background:**

In Ghana, pre-school-aged children (PSAC) are at risk of intestinal schistosomiasis and are living in need of praziquantel treatment. To better assess the infection burden within this vulnerable demographic group, we have provided a comparative assessment of the prevalence of *Schistosoma mansoni* in pre-school-aged children by urine circulating cathodic antigen (CCA) dipsticks, real-time PCR Taqman® faecal assays and Kato-Katz coproscopy.

**Methods:**

In all, 190 pre-school-aged children were sampled from three endemic communities (*viz*. Tomefa, Torgahkope/Adakope, and Manheam) around Weija dam, Southern Ghana. Fresh stool and urine samples were collected from all participants for diagnosis.

**Results:**

Among all the three communities, the urine-CCA assay recorded the highest prevalence values of 90.5% (95% CI 80.4–96.4), 87.9% (95% CI 76.7–95), and 81.2% (95% CI 69.9–89.6) in Tomefa, Torgahkope/Adakope, and Manheam respectively. Prevalence by real-time PCR was 50% (95% CI 35.5–64.5), 8% (95% CI 2.2–19.2) and 16.7% (95% CI 8.3–28.5), while by Kato-Katz was 55.6% (95% CI 42.5–68.1), 8.6% (95% CI 2.9–19) and 11.6% (95% CI 5.1–21.6) respectively. Children aged 1 year and over were found to be positive with the urine-CCA assay; by the ages of 3–4, over 50% were urine-CCA patent. The sensitivity and specificity of the POC-CCA dipsticks, when compared against the combined results of Kato-Katz/TaqMan results was found to be 84.1% (95% CI = 72.7–92.1) and 12.9% (95% CI = 6.6–22) respectively.

**Conclusions:**

We propose that the urine-CCA dipstick may be a useful rapid diagnostic tool to estimate the prevalence of intestinal schistosomiasis in PSAC, particularly in rapid identification of at-risk areas. However, our assessment has shown that it possible to record false positives when compared to combined Kato-Katz and qPCR results. To guide PSAC praziquantel treatment needs, we propose the urine CCA assay should be included in routine surveillance of intestinal schistosomiasis alongside other diagnostics such as Kato-Katz and urine filtration.

## Background

Schistosomiasis is a water-borne parasitic disease that is caused by chronic infection with flukes of the genus *Schistosoma.* The disease has serious public health implications, affecting 230 million people living in 54 countries [1, 2], with an estimated cost of eight million disability-adjusted life years [3].

In sub-Saharan Africa, *Schistosoma mansoni* is the predominant cause of intestinal schistosomiasis. The most commonly used method for diagnosing *S. mansoni* infection is by coproscopy with the Kato-Katz thick faecal smear which detects eggs shed in stool samples [4]. This assay has the advantage of being relatively cheap to perform, and therefore ideal for many resource poor settings where the disease occurs [1]. However, this assay may have low detection sensitivity in communities with low prevalence and intensity of infection [5]. With the expected expanded access to praziquantel [6–8], and a drive towards the interruption of transmission [8], there is the need for more sensitive diagnostic tools that can detect infections at low disease prevalence and intensity settings, also in currently excluded demographic groups such as pre-school-aged children (PSAC) in high endemic areas.

Another *S. mansoni* diagnostic assay is the schistosome DNA by real-time PCR Taqman® assay [9, 10]. Although this assay is based on eggs that have been shed into stool samples, the detection of DNA potentially gives this assay greater diagnostic sensitivity than the Kato-Katz assay [11–13]. Therefore, this assay can be used for disease surveillance in communities that are approaching interruption of transmission. This assay, however, has the limitation of being expensive and requiring highly skilled molecular biology technicians and equipment to operate. In addition, this assay is limited by day-to-day variations in *Schistosoma* egg production [14], and the low volume of faecal sample that is processed for DNA extraction.

Both adult male and female *Schistosoma* worms secrete Circulating Cathodic Antigen (CCA) in their vomitus [15, 16], therefore the detection of the CCA antigen, either in serum or urine, in an individual gives an indication of the presence of active infection(s) in a human host. The commercially available urine (point-of-care) POC-CCA diagnostic assay is in a lateral flow format (ICT International, Cape Town, South Africa) making it very user friendly. A major advantage of the urine CCA kit is the significantly minimal variation in the day-to-day production of this antigen by the adult worm, compared to variations in egg production and release by the adult female worm [17]. It may therefore be expected that urine-CCA assay will be more sensitive than the egg- based Kato-Katz [5, 11, 18–20] and real-time PCR Taqman® assays [11]. The urine CCA assay also has the advantage of detecting *S. mansoni* infections in urine, and not stool samples, which can be challenging to obtain from some cohorts within endemic communities such as PSAC. The urine-CCA assay, however, has the limitation of not being able to reliably detect *S. haematobium* infections, and may therefore be of limited use in areas of mixed infection.

Intestinal schistosomiasis infection has been detected in PSAC (viz. aged between 1 and 5 years) in Uganda [21, 22] and some other African countries [22]. From the current literature, there is no contemporary report on the prevalence of intestinal schistosomiasis among PSAC in Ghana - only Bosompem and colleagues [23], have reported on the prevalence of urinary schistosomiasis within this cohort in a endemic community in Ghana. Despite the recommendation [22] to include PSAC in routine praziquantel treatment activities, in Ghana the focus is still on school children, leaving out the at-risk PSAC cohort. In order to improve the chances of the inclusion of PSAC in the national control treatment strategies, there is the need for highly sensitive diagnostic assays to better assess disease burden within this cohort.

With the absence of a gold standard for the detection of *S. mansoni* infections [14], it is important for control programs or disease control interventions to be flexible, and apply the best diagnostic kit for a particular setting. In Ghana, routine field surveillance activities mainly utilize the Kato-Katz assay - a situation that can lead to an under estimation of disease prevalence and intensity, given the low diagnostic sensitivity of this assay under some disease settings. This could have negative impacts on the many years of control activities against the disease. In this work, we have applied the (point-of-care) POC-CCA diagnostic assay to detect *S. mansoni* infections in the urine of PSAC in Ghana. We evaluated this assay by comparing the CCA prevalence values with those from the stool based real-time PCR Taqman® and Kato-Katz assays.

## Methods

### Ethics

The Institutional Review Board (IRB) of the Council for Scientific and Industrial Research (research protocol number 003/CSIR-IRB/2016) and the Liverpool School of Tropical Medicine (research protocol number 16–044) granted ethical clearance for this study. This study is embedded with a larger study on the expanded access to praziquantel and albendazole in whole communities, see Campbell et al. (2018) and Cunningham et al. (in submission) for full details of sensitisation, enrolment and sampling procedures. Briefly, all participants were enrolled after community, household and individual sensitisation. Prior to sample collection, all parents or guardians signed a written consent form, and all participating PSAC assented. During the informed consent process, the importance of diagnosis in the determination of praziquantel treatment needs was explained to all parents and guardians. After sample collection, praziquantel was mass-administered in all communities to cover both study participants and non-participants using the World Health Organization recommended doze pole. There was also special care taken to ensure that all infected PSACs were treated.

### Study area

**Fig. 1:**
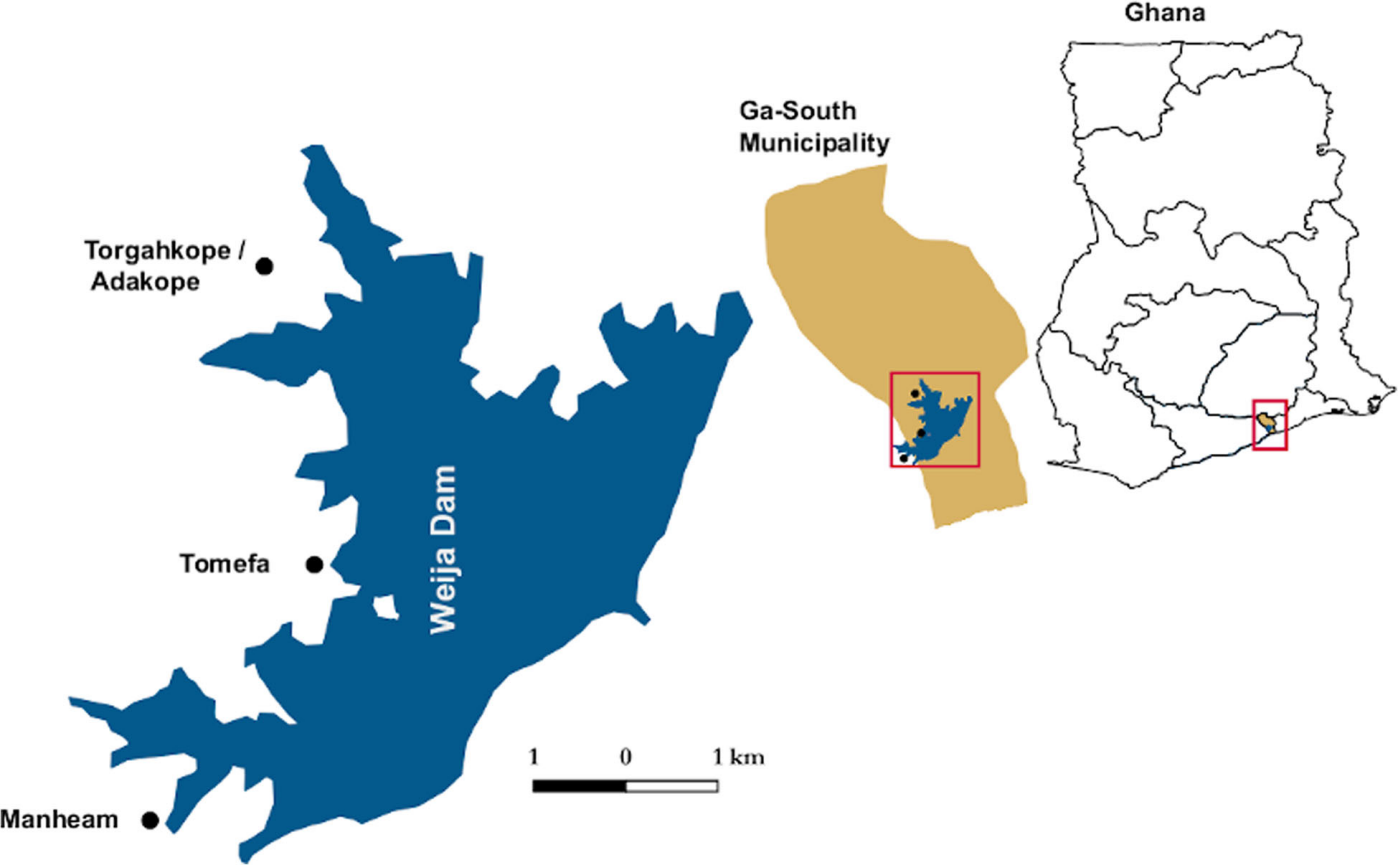
Studies sites, located in the Greater Accra region of Ghana, around the Weija dam

In the selection of study areas, reference was made to the National NTDs Control survey data that used the Kato-Katz assay to detect schistosomiasis infections. An initial set of twenty potential study areas were selected, and screened to arrive at a final set of three sites, which had schistosomiasis prevalence of at least 10%, based on school surveys performed by the Biomedical and Public Health Research Unit of CSIR. Additional selection criteria for the study sites included all-year-round accessibility by road and a low migration rate. All three sites were located around the Weija dam in Southern Ghana (Fig. 1). The sampled communities were Tomefa (longitude: − 0.37688, latitude: 5.57309), Torgahkope / Adakope (longitude: − 0.38176, latitude: 5.6055) and Manheam (longitude: − 0.39127, latitude: 5.55229). The Weija dam is the main reservoir from which water is sourced and treated for supply to more than half of the 2 million population of Accra, the capital city of Ghana. There are two distinct weather patterns within the endemic communities (viz. dry and rainy season). Because of the importance of the Weija reservoir, it never dries up during the dry season, so there is all-year-round availability of water in the dam, which could also be translated to the possibility of all-year-round transmission of schistosomiasis. For all the three sampled communities, fishing was the main of source of economic livelihood. Generally, the men did the fishing, whilst the women were involved in fish mongering. These fishing activities resulted in many water contact opportunities, which could be infective.

### Sample size calculation

This study is part of a larger intervention study by the COUNTDOWN Consortium [6]. Sample size calculation for this study was performed as described previously (6), using the formula below:

$$ n={Nz}^2\rho q/\left({E}^2\left(N-1\right)+{z}^2\rho q\right) $$

where: N = population size (approx. 1500 people); z = 1.96 (confidence level 95%); p = estimated prevalence (20%); q = 1-p; E = accuracy of estimation (0.03).

A final sample size of 658 people (including adults, school-age children, and PSAC) was computed for each community considering variations in age and sex.

### Sample collection

Children from ages 1 to 5 years were classified as PSAC. This study used samples provided by PSACs from whole community sample collection that was carried out in the second quarter of 2017 as part of a larger COUNTDOWN Consortium study [6] in the three study sites (Fig. 1). In all, 190 PSAC from the three sampling areas participated in this study. All study participants were registered and given a unique identification code, which were then used to label empty urine and stool containers for sample collection. During the informed consent process, it was requested that participants present the first urination and bowel movement of the day as samples for parasitological analyses. Each of the 190 PSACs provided single urine and stool samples.

### Laboratory procedures

Duplicate Kato-Katz thick smear slides were prepared for each stool sample (Sterlitech) as done elsewhere [11, 24]. Stool samples were assessed (using Olympus model CH and Boshida BD-SW30 microscopes) at × 400 magnification by two different experienced technicians. As a quality control check, the lead technician examined 10% of the Kato-Katz slides in order to confirm positive or negative results. All urine samples were tested for *S. mansoni* using the CCA test kits (ICT International, Cape Town, South Africa) following the manufacturer’s protocol. In brief, all POC-CCA test reagents were equilibrated to room temperature prior to use. A straw dropper was used to transfer two drops (each drop is equivalent to 40 uL) of urine from the urine sampling container to the circular receptacle on the POC-CCA cassette. After 20 minuntes, the POC-CCA test were scored as follows, negative (−), light/trace band (+), medium band (++) and heavy band (+++). The visual assessment was carried out independently by two trained technicians using a visual guide to help inform their assessment (Additional file 1). In addition, the samples were tested for *S. mansoni* infection using a real-time PCR Taqman® assay. In brief, DNA was isolated from each stool sample using the QIAamp DNA mini kit (QIAGEN, Hilden, Germany) following the manufacturer’s protocol. The phocin herpes virus 1 (PhHV-1) was used as an internal control for all DNA extractions [10, 25]. For the real-time PCR Taqman® assay, the internal-transcribed-spacer-2 sequence of the *S. mansoni* ribosomal RNA gene (GeneBank Accession Number: AF503487) was targeted [10] as done elsewhere for real-time PCR diagnosis of schistosome parasite infections [10, 25]. Each PCR reaction mix (20 μL total volume), contained 200 nM of primers (SP: 5′-GGT CTA GAT GAC TTG ATY GAG ATG CT-3′; ASP 5′-TCC CGA GCG YGT ATA ATG TCA TTA - 3′), 2 μL of DNA, 100 nM of Taqman probe (5′-TGG GTT GTG CTC GAG TCG TGG C – 3′), and 12.5 μL of iQ™ Supermix (Bio-Rad Laboratories Inc., California, USA). Each run was performed on the Rotor Gene thermocycler (QIAGEN, Hilden, Germany) using the following reaction conditions: initial incubation at 95 °C for 3mins, followed by 50 cycles of 95 °C for 15 s, 60 °C for 30s, 72 °C for 30s, and a final incubation step of 72 °C for 2mins. All qPCR runs were performed on 96-well plates, which also had a positive control of *S. mansoni* DNA extracted from the Liverpool of Tropical Medicine collection of whole adult worms. A sample was scored as positive if the internal control amplified, and the *S. mansoni* specific primers resulted in amplification which had the same melt peak profile as the *S. mansoni* positive control. All qPCR runs were accompanied by negative controls of sterile double distilled water. After all qPCR runs, 31 of the DNA samples did not show amplification of the internal PhHV-1 control, and were thus excluded from the study leaving a total of 160 samples for analyses.

### Data management and statistical analysis

Results of the parasitological assessments were recorded on proforma data sheets. The data were then double entered in Microsoft Excel prior to statistical analyses. The R programming software, version 3.2.2 [26] was used to generate summary tables and figures. The same software was used to determine the 95% confidence intervals for prevalences employing the exact method, and also perform Fisher’s exact test to determine differences between prevalences in relation to diagnostic techniques and communities of origin. The Kappa statistic was used to give a quantitative agreement between the CCA results and the composite gold standard (Kato-Katz and qPCR). Interpretation of Kappa values, are as follows: < 0 less than chance, 0.01–0.20 slight agreement, 0.21–0.40 fair agreement, 0.41–0.60 moderate agreement, 0.61–0.80 substantial agreement and 0.81–0.99 almost perfect agreement [27].

## Results

The numbers of PSAC from the three communities who participated in this study differed between tests and site, due to the availability of samples. The following are the numbers of samples processed per diagnostic assay from each community: Tomefa 63 samples were screened using both Kato-Katz and POC-CCA, 50 were screened using TaqMan qPCR. From Torgahkope/ Adakope 69 samples were processed with both Kato-Katz and POC-CCA, 60 were screened with TaqMan qPCR. A total of 58 samples from Manheam were screened using Kato-Katz and POC-CCA, 50 of these samples were also screened with the TaqMan qPCR.

### Evaluation of the prevalence of *S. mansoni* determined by the urine CCA, real-time PCR Taqman® and Kato-Katz assays

**Fig. 2:**
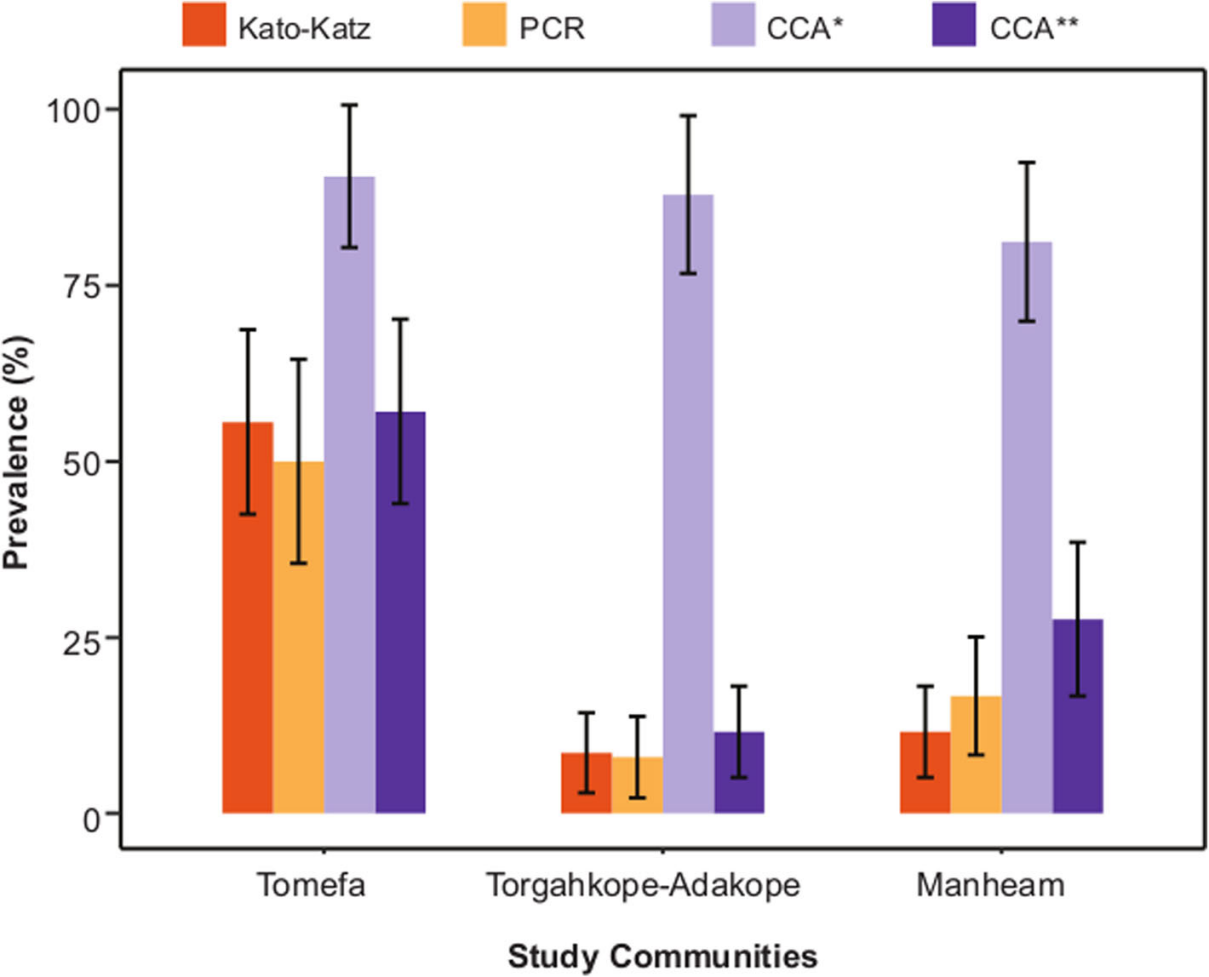
Positive rates across the three sampling sites for Kato-Katz, qPCR and CCA results, *with and **without faint/trace bands

For the purpose of evaluation, the prevalences of *S. mansoni* infection in the PSAC, as determined by the urine CCA, real-time PCR Taqman®, and Kato-Katz assays, are presented in Fig. 2 and Table 1. The results of the POC-CCA were evaluated to both include and exclude light/trace bands. In both instances the POC-CCA identified the largest number of positives from all communities with Tomefa identified as having the highest prevalence at 90.5% (57/63; 95% CI = 80.4–96.4) when light/trace bands are counted and 57.1% (36/63; 95% CI = 44.0–69.5) without the light/trace results. The POC-CCA, when including light/trace results, identified Torgahkope/Adakope as having the second highest prevalence, 87.9% (51/58; 95% CI = 76.7–95), however when the light/trace results were excluded Manheam was identified as having the second highest prevalence, 27.6% (16/58; 95% CI = 16.7–40.9). Manheam was identified as having the lowest prevalence of *S. mansoni* when the light/trace results were included, 81.2% (56/69; 95% CI = 69.9–89.6) and Torgahkope/Adakope when these were excluded, 11.6% (8/69; 95% CI = 6.3–25.8). The results of both the Kato-Katz and real-time PCR reflected the results of the POC-CCA, when the light/trace results were excluded. Once more Tomefa was identified as having the highest prevalence out of three communities by Kato-Katz, 55.6% (35/63; 95% CI = 42.5–68.1) and qPCR, 50% (25/50; 95% CI = 35.5–64.5), followed by Manheam; Kato-Katz 11.6% (8/69; 95% CI = 5.1–21.6), Taqman PCR 16.7% (10/60; 95% CI = 8.3–28.5). Torgahkope/Adakope was identified as having the lowest prevalence by Kato-Katz, 8.6% (5/58; 95% CI = 2.9–19) and real-time PCR, 8% (4/50; 95% CI = 2.2–19.2).

**Table 1:** Prevalence of the *S. mansoni* infections determined by the three diagnostic assays across all study areas. For all test assays; n represents the total number of participants; Pos = number positive; and Prev (95% CI) = prevalence with 95% confidence interval

Study area	Diagnostic assay											
	Kato-Katz			PCR			POC-CCA^a^			POC-CCA^b^		
	***n***	Pos	Prev (95% CI)	***n***	Pos	Prev (95% CI)	***n***	Pos	Prev (95% CI)	***n***	Pos	Prev (95% CI)
**Tomefa**	63	35	55.6 (42.5–68.1)	50	25	50 (35.5–64.5)	63	57	90.5 (80.4–96.4)	63	36	57.1 (44–69.5)
**Torgahkope-Adakope**	58	5	8.6 (2.9–19)	50	4	8 (2.2–19.2)	58	51	87.9 (76.7–95)	69	8	11.6 (5.1–21.6)
**Manheam**	69	8	11.6 (5.1–21.6)	60	10	16.7 (8.3–28.5)	69	56	81.2 (69.9–89.6)	58	16	27.6 (16.7–40.9)
**Total**	**190**	**48**	**25.3 (19.3–32.1)**	**160**	**39**	**24.4 (17.9–31.8)**	**190**	**164**	**86.3 (80.6–90.9)**	**190**	**60**	**31.6 (25–38.7)**

^a^With light/trace results

^b^Without light/trace results

In all three sites, the POC-CCA (with trace results) gave the highest prevalence of intestinal schistosomiasis infection (Fig. 2). This was followed by POC-CCA prevalences without trace results, although the confidence intervals overlap with those of the Kato-Katz and real-time PCR. Indicating that without trace results the POC-CCA results can be similar to results from Kato-Katz and real-time PCR.

**Fig. 3:**
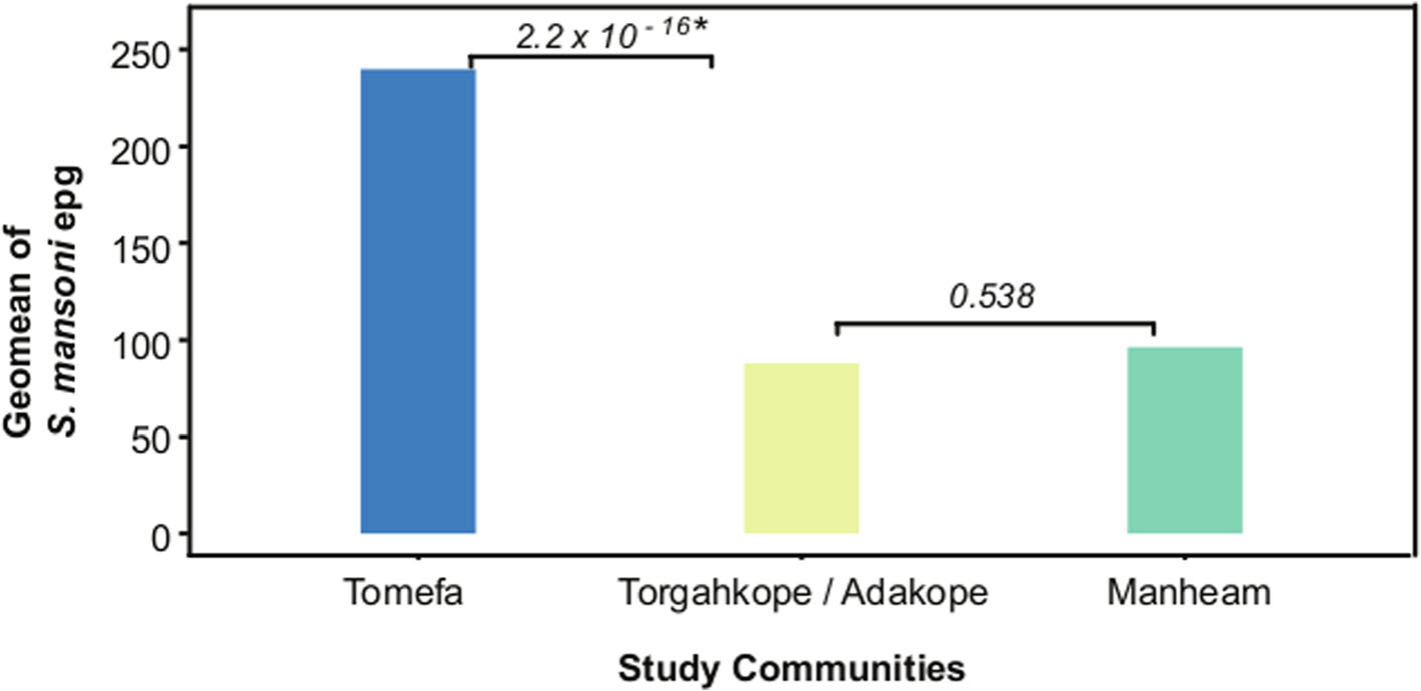
Geometric mean of *S. mansoni* epg numbers across the three study sites

The geometric mean of *S. mansoni* egg per gram (epg) of stool samples per community that were determined by the Kato-Katz assay is presented in Fig. 3. Tomefa recorded the highest geometric mean epg of 239.78, which was significantly different from the 87.99 of Torgahkope / Adakope (*P =* 2.2 × 10^− 16^), and the 96.36 of Manheam (*P =* 5.1 × 10^− 15^). There was however, no statistically significant difference between the geometric epg of Torgahkope / Adakope and Manheam (*P =* 0.538).

### Sensitivity, specificity and kappa score of urine CCA assay against a composite gold standard of real-time PCR and Kato-Katz assay positives

In the absence of a robust gold standard for the detection of *S. mansoni* infections [14], the sensitivity and specificity of the urine CCA assay was determined using the combination of positive results from the real-time PCR Taqman® and Kato-Katz assays as a composite gold standard as recommended elsewhere [28, 29]. This approach was adopted due to the consistently high prevalence values of the CCA compared to the PCR Taqman® and Kato-Katz assays. When faint/trace bands were included, the urine CCA assay had a sensitivity of 84.1% (95% CI = 72.7–92.1) and, 36.5% (95% CI = 24.7–49.6) when faint/trace bands were excluded. The specificity for the CCA test was 12.9% (95% CI = 6.6–22) and 76.5% (95% CI = 66–85) when faint/trace bands were included and excluded, respectively (Table 2). The Kappa values for the CCA showed less than chance agreement (− 0.03) with the composite gold standard when faint/trace bands are included and a slight agreement (0.14) when faint/trace bands are excluded (Table 2).

**Table 2:** Sensitivity, specificity and Kappa scores of the POC-CCA test versus combined Kato-Katz and PCR results

		Kato-Katz + PCR		Sensitivity	Specificity	Kappa Score
		Pos	Neg	(95% CI)	(95% CI)	
**CCA**^a^	**Pos**	53	74	84.1 (72.7–92.1)	12.9 (6.6–22)	−0.03
	**Neg**	10	11			
**CCA**^b^	**Pos**	23	20	36.5 (24.7–49.6)	76.5 (66–85)	0.14
	**Neg**	40	65			

^a^with trace bands

^b^without trace bands

### Age and positive status

The prevalences of *S. mansoni* infections among PSAC (with age stratification), as determined by the three diagnostic assays, are presented in Table 3. In all the study communities, the urine CCA assay produced the highest number of positives in PSAC 0–1 years of age. The real-time PCR Taqman® was also able to detect infection in this group in Manheam (Table 3). The urine CCA assay also gave the highest prevalence in all the communities (Table 3). It is also clear that in the latter ages (4–5 years) of the PSAC, all three assays were able to detect infections in all the communities. Our findings show early exposure to infection in these endemic areas, and the need for education and behaviour change, since PSAC do not have to perform mandatory economic and lifestyle activities that will expose them to infection from the dam. A common source of infection among the PSAC will be through water carried in pans from the dams for their bathing. With appropriate education, water for bathing can be stored and used after 3 days, when the cercariae are less likely to be infective [1]. Our findings also reveal PSAC treatment needs, and the need for an infant formula for praziquantel.

**Table 3:** Positives stratified by age for Kato-Katz, qPCR and CCA, for each community: Tomefa, Torgahkope/Adakope and Manheam. For all test assays; n represents the total number of participants; Pos = number positive; and (Prev; 95% CI) = prevalence with 95% confidence interval

Age	Kato-Katz		PCR		CCA^a^		CCA^b^	
	***n***	Pos (Prev; 95% CI)	***n***	Pos (Prev; 95% CI)	***n***	Pos (Prev; 95% CI)	***n***	Pos (Prev; 95% CI)
**Tomefa**								
0–1	2	0	2	0	3	3 (100; 29.9–100)	3	2 (66.7; 9.4–99.2)
2–3	42	24 (57.1; 41–72.3)	35	13 (37.1; 21.5–55.1)	42	37 (88.1; 74.4–96)	42	24 (57.1; 41–72.3)
4–5	13	9 (69.2; 38.6–90.9)	11	6 (54.5; 23.4–83.3)	13	12 (92.3; 64–99.8)	13	7 (53.8; 25.1–80.8)
**Torgahkope/Adakope**								
0–1	1	0	1	0	1	1 (100; 2.5–100)	1	0
2–3	37	6 (16.2; 6.2–32)	28	5 (17.9; 6.1–36.9)	37	32 (86.5; 71.2–95.5)	37	6 (16.2; 6.2–32)
4–5	19	2 (10.5; 1.3–33.1)	14	2 (14.3; 1.8–42.8)	19	17 (89.5; 66.9–98.7)	19	2 (10.5; 1.3–33.1)
**Manheam**								
0–1	3	0	3	1 (33.3; 0.8–90.6)	3	3 (100; 29.2–100)	3	2 (66.7; 9.4–99.2)
2–3	34	2 (5.9; 0.7–19.7)	32	3 (9.4; 2–25)	34	28 (82.4; 65.5–93.2)	34	6 (17.6; 6.8–34.5)
4–5	24	2 (8.3; 1–27)	22	4 (18.2; 5.2–40.3)	25	19 (76; 54.9–90.6)	25	7 (28; 12.1–49.4)

^a^With light/trace results

^b^Without light/trace result

## Discussion

In this study, we evaluated the urine CCA assay against the real-time PCR Taqman® and Kato-Katz assays for the detection of *S. mansoni* in three endemic communities in southern Ghana. PSAC are an important cohort within endemic communities [30] because they carry low parasite densities due to their relatively short period of exposure to infection. This requires that very sensitive diagnostic assays must be applied to limit the risk of under-estimating disease prevalence within this cohort. It is the first occasion that intestinal schistosomiasis has been observed to be common in Ghanaian PSAC.

In all the three study communities, the urine CCA assay recorded the highest prevalence values among the three assays (Fig. 2). A review of multiple studies by Kittur and colleagues [5] revealed that when the Kato-katz prevalence is less than 20%, the urine CCA prevalence is 3 to 6 fold higher. In addition, a recent study found that urine CCA prevalence could be up to 8 fold higher than Kato-katz prevalence in low endemicity settings [31]. We observed a similar trend in Manheam, which had a Kato-Katz prevalence of 7% (Fig. 2); and Torgahkope/Adakope, which had a Kato-Katz prevalence of 21% (Fig. 2). Our findings confirm reports that the urine CCA assay is capable of identifying more samples as positive compared to Kato-Katz assay [5, 11, 19, 31–33]. This study also confirmed (Fig. 2) that the urine CCA assay can have a significantly higher diagnostic sensitivity than the real-time PCR Taqman® assay [11]. However, it also possible that the faint bands are false positives as similar findings have been found before in other comparative diagnostic studies [34]. This feeds into the controversy around the inclusion or exclusion of faint/trace bands [35]. Based on our findings and field experience, not all faint bands are false positives, so cannot be wholly ignored. Other studies have sought to optimise the CCA-test by developing field friendly urine-concentration methods [36] or improving the resolution of the scoring system [37].

A possible explanation for the significantly higher urine CCA prevalence could be due to the secretion of the antigen by both sexes of the adult *S. mansoni* fluke [15, 16]. This enables the detection of active infections when only viable adult male flukes live in a host, which is a likely infection status for PSAC who usually have low exposure rates to infective agents of schistosomiasis. In the absence of a reproductively active adult female fluke, the real-time PCR Taqman® and Kato-Katz assays, which relies on shed eggs, will diagnose an individual as negative despite a possible infection with adult male flukes.

Another possible explanation for the high urine CCA prevalence is that, unlike the real-time PCR Taqman® and Kato-Katz assays that may be limited by low infection density of adult female worms (and a likely low density of shed eggs), the urine CCA assay is able to detect low densities of the CCA antigen that has been released by active adult fluke infections. In addition there is an advantage of significantly lower day-to-day variations in the secretion of the CCA antigen compared to variations in egg production by the adult female flukes [1, 17, 38]. This could make the day and time of sampling an important confounding factor for the findings of the real-time PCR Taqman® and Kato-Katz assays.

The Kato-Katz prevalence, was 8.6% in Torgahkope/Adakope and 11.6%, in Manheam (Table 1). For the Manheam, the real-time PCR Taqman® higher than the Kato-Katz prevalence (Table 1). A similar trend was reported elsewhere [11], indicating that the real-time PCR Taqman® can be more sensitive than Kato-Katz assay. Despite this, the Kato-Katz prevalence was higher than qPCR in Tomefa. This may be a challenging observation to explain, but a plausible explanation could be low intensities of egg in stool samples that could not be detected by real-time PCR Taqman® assay because of high concentration of background DNA from other biological materials in stool samples. It is also possible that the population of *Schistosoma* flukes in Tomefa and Torgahkope / Adakope exhibit a high rate of genetic variation leading to sub-sampling during real-time PCR Taqman® reaction. Therefore, there may be the need to investigate genetic differentiation in some field isolates of *Schistosoma* sp., given that extensive genetic variation has been recorded in some parasite isolates [39].

In the control of schistosomiasis, determining the minimum age of when infection can be acquired within an endemic community can be very useful in designing intervention strategies [21]. However due to the low numbers of individuals in the different age groups, the results from an age stratified approach resulted in very large confidence intervals for the majority of cases meaning little significant difference could be detected, if any at all (Table. 3).

## Conclusion

In conclusion, the data presented here are consistent with the findings of previous reports that indicated that the urine CCA assay is more sensitive than the real-time PCR Taqman® and Kato-Katz assays [11] but could be limited by poor specificity, resulting in high numbers of false positives [34]. This could be tackled by having a critical view of faint/trace bands. Issues regarding the inclusion of faint/trace bands are well understood in the community and steps have been taken to overcome this short-fall of the CCA assay by pre-treating the urine and having a better evaluation protocol for reading the results. The urine CCA dipstick remains a viable assay for routine surveillance activities and highlights its use for routine surveillance of intestinal schistosomiasis in PSAC in conjunction with MDA campaigns. This CCA assay can be used alongside commonly used assays such as the Kato-Katz and urine filtration assays since they still remain viable. With regards to sample collection from PSAC, our field experience thought us that it was more challenging to obtain stool samples from PSAC for parasitological analyses. Rather, it was significantly less challenging to obtain urine samples from the PSAC. This means that with a single urine sample, control programs can detect both intestinal and urinary schistosomiasis status by CCA and urine filtration assays respectively.

## Supplementary information

**Additional file 1.** Visual assessment guide used by two trained technicians for scoring POC-CCA results. The test was scored as follows, negative (−), light/trace band (+), medium band (++) and heavy band (+++).

## Data Availability

The Dataset used and analysed here are available from the corresponding author on reasonable request.

## Abbreviations

CCA: Circulating cathodic antigen
CI: 95% confidence interval
epg: egg per gram
PCR: Polymerase chain reaction
PSAC: Pre-school -aged children
