# Supplementary figures and images for: Detecting Schistosoma mansoni infections among pre-school-aged children in southern Ghana: a diagnostic comparison of urine-CCA, real-time PCR and Kato-Katz assays
Source: BMC Infect Dis. 2020 Apr 22;20:301. doi: 10.1186/s12879-020-05034-2 (PMC7178570; doi:10.1186/s12879-020-05034-2)

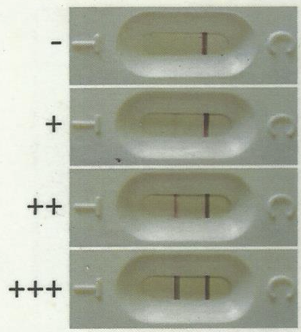

Supplement: Supplementary file 1 — Additional file 1. Visual assessment guide used by two trained technicians for scoring POC-CCA results. The test was scored as follows, negative (−), light/trace band (+), medium band (++) and heavy band (+++). [file 12879_2020_5034_MOESM1_ESM.tif]
